# Quantitative Evaluation of Mechanical Properties of Hydrogen Transmission Pipelines Based on Weak Magnetic Detection

**DOI:** 10.3390/s25123778

**Published:** 2025-06-17

**Authors:** Siyang Wang, Xianglong Sun, Xingyuan Miao, Haimu Ye

**Affiliations:** 1 College of New Energy and Materials, China University of Petroleum (Beijing), Beijing 102249, China; 2 College of Mechanical and Transportation Engineering, China University of Petroleum (Beijing), Beijing 102249, China; 3 School of Mechanical Electronic Engineering, China University of Mining and Technology (Beijing), Beijing 100083, China

**Keywords:** weak magnetic detection, magnetic characteristics, mechanical properties, model modification, hydrogen transmission pipelines

## Abstract

With the rapid development of the hydrogen energy industry, long-distance hydrogen transportation based on natural gas pipelines has emerged as a crucial technique. However, exposure to a hydrogen environment can lead to the degradation of pipeline mechanical properties, resulting in hydrogen corrosion, which may increase the risk of pipeline failure. Consequently, it is crucial to evaluate the mechanical properties of pipeline steel under a hydrogen environment to ensure pipeline safety. In this paper, hydrogen corrosion experiments for X80 pipeline steel are carried out with varying hydrogen charging times. Through tensile fracture experiments and weak magnetic detection technology, the effects of defects and hydrogen concentration on the stress–strain characteristics and magnetic signal characteristics of X80 steel are investigated. Based on the correlation level, the quantitative relationships between hydrogen concentration, magnetic signal characteristics, and mechanical properties are established, and the sparrow search algorithm (SSA) is utilized to modify these quantitative relationships. The results indicate that with the increase in defect depth, the magnetic signal characteristics gradually increase. With the increase in defect diameter, these parameters gradually decrease. The modified quantitative relationships provide the accurate assessment of the ultimate bearing capacity, yield strength, and ultimate tensile strength, with average relative errors of 7.91%, 3.15%, and 2.04%, respectively. This study provides a theoretical basis for ensuring the safe transportation of hydrogen transmission pipelines.

## 1. Introduction

Pipelines are the primary means of transportation of oil and natural gas resources [1,2]. Hydrogen energy transportation is crucial for the sustainable development of the hydrogen energy industry chain. Using natural gas pipelines for hydrogen transportation is a large-scale and long-distance method for hydrogen energy transmission [3,4]. However, hydrogen molecules in the pipeline medium can adsorb onto the pipeline inner wall, decompose into hydrogen atoms, and subsequently penetrate pipeline material [5], which can lead to a loss of toughness or hydrogen-induced cracks and even pipeline fracture failure. Due to its exceptional strength and toughness, X80 steel has been extensively utilized in natural gas pipeline applications. Consequently, X80 steel is considered a promising material for hydrogen blending pipelines [6], but hydrogen corrosion can decrease the mechanical properties of this pipeline steel, increasing the risk of pipeline failure. Therefore, its study is vital for the accurate detection of hydrogen corrosion defects and the assessment of the mechanical properties of X80 steel.

Non-destructive testing (NDT) mainly includes ultrasonic testing, X-ray testing, eddy current testing, magnetic flux leakage (MFL), weak magnetic detection [7], etc. Ultrasonic testing has a high detection speed and wide range of applicable conditions [8]. However, this technology requires a couplant during the detection process. X-ray testing offers strong penetration capabilities and is suitable for various materials [9] but necessitates radiation protection during the detection process. Eddy current testing is characterized by its high sensitivity, which can detect defects with a small size [10], but it is difficult for deep defect detection and accurately quantifying their sizes. MFL is advantageous in surface or near-surface defect detection, providing fast detection speeds and good visualization. However, MFL technology requires a strong magnetic field, which makes the detection devices bulky and inefficient, and it is susceptible to interference from external excitation magnetic fields [11]. Weak magnetic detection technology exhibits high sensitivity to defects and stress concentration zones. In contrast to other NDT methods, weak magnetic detection does not necessitate an external excitation magnetic field [12,13], nor does it require any surface pre-treatment procedures. This characteristic results in simpler equipment requirements and more convenient operational processes.

In recent years, scholars have conducted research on defect and stress detection based on weak magnetic technology. The magnetic dipole method was firstly introduced by Roskosz and Bieniek [14], who incorporated residual magnetic field components into Maxwell’s equations to establish a relationship between residual stress and magnetic field derivatives. Venkatachalapathi et al. [15] investigated the weak magnetic signals in the load regions of ferromagnetic materials, elucidating the variation patterns of these signals in response to external loads. Jung et al. [16] studied the relationship between stress and applied magnetic fields, deducing the underlying principles behind the generation of weak magnetic signals at crack locations. Yang et al. [17] analyzed weld and non-weld samples of Q345 materials through tensile and compressive fatigue tests, discovering that weak magnetic characteristics could accurately predict the fatigue life of these samples. Liu et al. [18] validated the characteristics of weak magnetic signals for internal pipeline stress inspection, establishing a magneto-mechanical equivalent model with a balanced magnetic field. The Boltzmann lift-off correction factor was proposed, and the adaptability of the equivalent model could reach 94%. Additionally, magnetic force calculations were incorporated into Maxwell’s equations to develop a mathematical model for triaxial weak magnetic stress detection. The results demonstrated that weak magnetic signals demonstrated an exponential decay trend during propagation, with the highest recognition accuracy for axial signals. The circumferential and radial components exhibited strong capabilities to avoid false negatives and false positives, respectively. Li et al. [19] constructed a weak magnetic detection model for buried pipeline defects based on the magnetic charge theory, where the influences of model parameters and defect characteristics on the detection signals are investigated. This numerical method offers an innovative approach for forward models of weak magnetic detection in buried pipelines. Hu et al. [20] used the finite element method to solve magnetic signal intensity at surface defects in titanium alloys, discovering that a critical defect width of 0.6 mm is detectable using weak magnetic technology. Le et al. [21] extracted difference curves of weak magnetic signals from the same specimen under varying tensile loads, finding that the first-order difference curve of magnetic signals in stress concentration areas was smooth and continuous. Liu et al. [22] proposed a quantitative model for calculating the internal inspection signals of non-uniform magnetic charge; the influences of crack depth, crack width, and internal pressure variations on weak magnetic signals are studied; and the theoretical results are verified through extensive experiments.

The above literature reviews have indicated that weak magnetic detection technology can be used to assess the defects and stresses for oil and natural gas pipelines. However, current studies are limited for hydrogen transmission pipelines due to insufficient research on weak magnetic characteristics under stress loads and hydrogen experiments. The coupling effects of hydrogen, defects, and stress can affect the magnetic signal characteristics. Additionally, there are few studies on the quantitative evaluation of the mechanical properties of hydrogen transmission pipelines.

In this study, a hydrogen corrosion experiment is conducted on X80 pipeline steel at different hydrogen charging times. Tensile fracture experiments and weak magnetic detection technology are employed to analyze the influences of defects and hydrogen concentrations on both stress–strain characteristics and magnetic signal characteristics. Based on the correlation level, the quantitative relationships between the hydrogen concentration, magnetic signal characteristics, and mechanical properties are established. Finally, in order to evaluate the mechanical properties of X80 steel with different defects, the sparrow search algorithm (SSA) is utilized to modify the quantitative relationships.

## 2. Experiments

### 2.1. Theoretical Foundation of Magnetic Changes for Hydrogen-Charged Pipeline Steel

**Figure 1:**
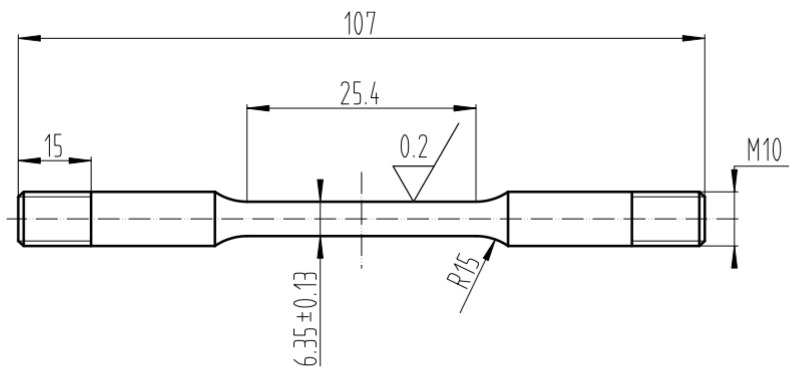
The size and shape of the tension specimen [mm].

In this study, X80 pipeline steel is selected as the research subject, which is a high-strength steel primarily used in long-distance oil and natural gas pipelines. The size and shape of the tension specimen are shown in Figure 1, while the chemical composition of X80 steel is listed in Table 1.

**Figure 2:**
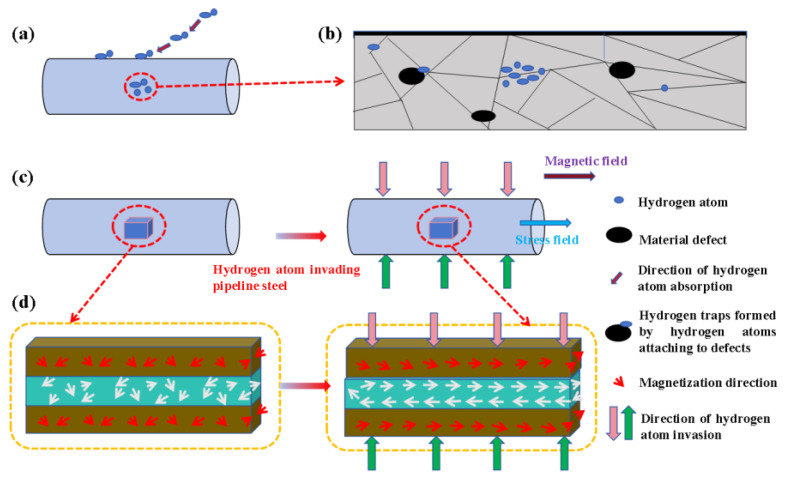
The process of magnetic change in pipeline steel in hydrogen environments: (**a**) hydrogen atoms adsorbed into the interior of pipeline steel; (**b**) hydrogen atom invasion and diffusion inside the material; (**c**) pipeline steel before and after the external application of a stress field; (**d**) changes in the direction of magnetization in the structure of the magnetic domains before and after the invasion of the hydrogen atoms into the pipeline steel.

As a ferromagnetic material, pipeline steel exhibits distinct magnetic and mechanical properties when subjected to external forces. Hydrogen atoms can be readily captured by defects within the steel and accumulate internally due to dislocation effects, which influences the magnetic domain structure and alters the material’s magnetic characteristics. According to the Hydrogen Enhanced Local Plasticity (HELP) theory [23], hydrogen atoms diffuse through lattice vacancies, with their movement being influenced by temperature and crystal structure. Stress-induced hydrogen diffusion results in an accumulation of hydrogen in high-stress regions, thereby affecting the magnetoelastic properties of the material [24,25]. According to the theory of hydrogen embrittlement degradation (HEDE), hydrogen atoms tend to accumulate at grain boundaries and other microstructural features within materials. This accumulation weakens the bonding strength of the materials, thereby reducing their ductility and increasing their susceptibility to brittle fracture [26]. According to the theory of hydrogen-induced cracking (HIC), hydrogen diffuses through the material and accumulates at defect sites, such as crack tips and voids. This accumulation leads to localized high pressure, which, in turn, facilitates both the initiation and propagation of cracks [27]. According to the theory of hydrogen-induced stress corrosion cracking (H-SCC), the simultaneous influence of mechanical stress and a corrosive environment leads to the penetration of hydrogen atoms into the metal’s interior. This process results in material embrittlement and facilitates the propagation of cracks [28]. The interplay between hydrogen diffusion and alterations in the magnetic domain structure significantly influences both the mechanical and magnetic properties. When pipeline steel is subjected to stress, Figure 2 illustrates the changes in magnetic properties under the combined influence of stress and hydrogen atoms. As external hydrogen penetrates the material, these atoms continuously migrate at metal defects, further influencing its magnetic attributes. When pipeline steel experiences external stress, its magnetic properties undergo modifications as a result of the synergistic effects of stress and hydrogen presence. The extent of hydrogen atom infiltration is assessed through variations in the material’s magneto-mechanical properties, thus establishing a coupling relationship among hydrogen concentration, magnetism, and mechanical properties.

### 2.2. Hydrogen Corrosion Experiment

**Figure 3:**
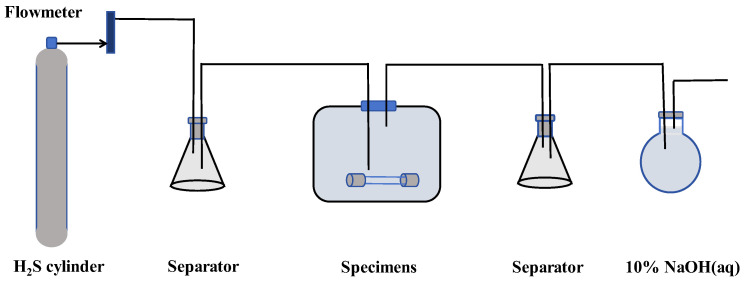
Schematic diagram of hydrogen corrosion process.

Hydrogen corrosion is a complex electrochemical process that involves hydrogen adsorption and permeation, hydrogen dissolution, hydride formation, hydrogen blistering, hydrogen embrittlement, and accelerated corrosion. To simulate the hydrogen experiment, the H_2_S corrosion method is used to accelerate the hydrogen corrosion process of the specimens [29]. The schematic diagram of the hydrogen corrosion process for X80 steel specimens is shown in Figure 3. To ensure the accuracy and consistency of the analytical results, the defect shape is transformed into an equivalent semi-elliptical defect. In most instances, the hydrogen corrosion failure mode associated with hole defects aligns with that of the equivalent semi-elliptical defects. Therefore, a total of 16 specimens with a length of 107 mm are prepared, which are divided into four groups based on different hole defects (no defects, 1 mm diameter with 1 mm depth, 1 mm diameter with 2 mm depth, and 2 mm diameter with 1 mm depth). Each group consists of four specimens with different hydrogen charging times, and X80 steel specimens with different defects are shown in Table 2. Before the hydrogen corrosion experiment, both sides of the target corrosion area are wrapped with insulating tape to prevent corrosion in other areas. At the end of the hydrogen corrosion experiment, the residue in the corrosion area is cleaned, and the X80 steel specimens are dried.

### 2.3. Tensile Fracture Experiment

**Figure 4:**
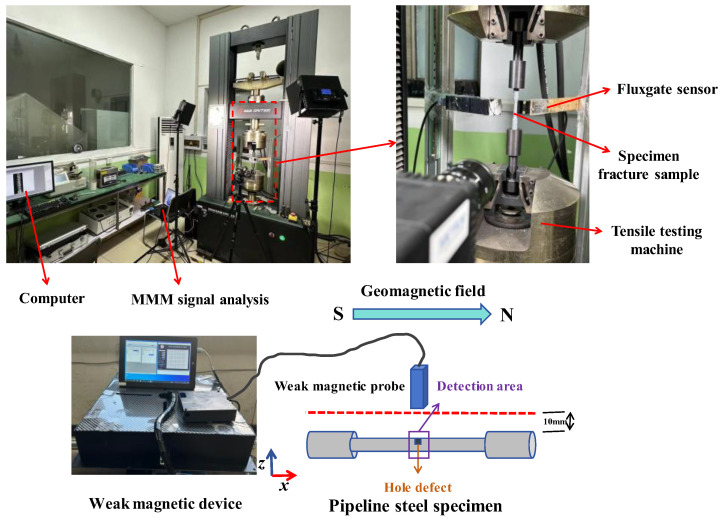
The tensile fracture experimental setup of the X80 steel specimens.

A total of 48 specimens are subjected to uniaxial tensile fracture experiments on a WAW-1000 electro-hydraulic servo tensile testing machine. The tensile fracture experimental setup of the X80 steel specimens is illustrated in Figure 4. Initially, the tensile testing machine is unloaded, and the loading speed is set to 1 mm/min. Then, the specimens are placed on the machine, where the weak magnetic signals and stress–strain curves are collected simultaneously. The tensile fracture experiment finishes when the specimens fracture.

Weak magnetic detection technology is based on the environment of geomagnetic field, and no external excitation magnetic field is required, making the operation convenient and efficient. Under the combined action of stress and geomagnetic field on ferromagnetic materials, high stress energy will exist at the location where the defects occur, resulting in magnetostrictive and reverse magnetostrictive effects. Under the influence of stress, the magnetic domains within the material tend to align in a direction that minimizes free energy [30,31]. During the magnetization process, the displacement of magnetic domain walls occurs, which can result in the formation of stable dislocation slip bands. The increase in magnetoelastic energy effectively counterbalances any rise in stress energy. This phenomenon is influenced by the Barkhausen effect, so it is irreversible. Consequently, even if external loads are subsequently removed, residual stresses persist due to frictional effects within the metal matrix at these locations. As a result, there remains an enduring state of magnetization characterized by a residual magnetic field. The fluxgate sensor is used to measure the weak magnetic signals in this experiment. The lift-off value from the measuring point to the surface of the specimens is 10 mm. The detection direction of the fluxgate sensor is oriented from the S-pole to the N-pole of the geomagnetic field.

## 3. Experimental Results

### 3.1. Hydrogen Corrosion Results

**Figure 5:**
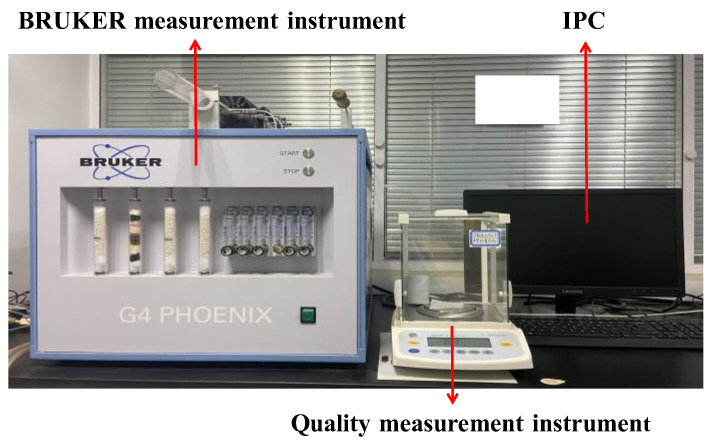
The experimental setup of hydrogen concentration measurement.

**Figure 6:**
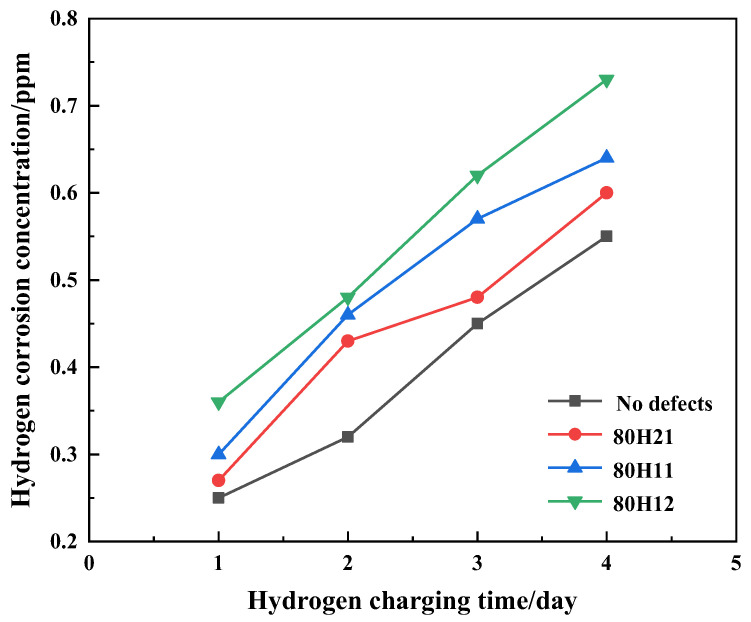
The results of hydrogen concentration of X80 steels with different defects.

The specimens after tensile fracture are input into the BRUKER measurement apparatus for testing the hydrogen concentration, which allows for the determination of hydrogen concentration at different hydrogen charging times, as depicted in Figure 5. Figure 6 illustrates the results of the hydrogen concentrations of X80 steels with different defects. The experimental results indicate a linear correlation between the hydrogen charging time and hydrogen concentration. Moreover, it is evident that the hydrogen concentration increases with increasing defect depths. With the increase in defect diameters, the hydrogen concentration decreases.

### 3.2. Stress–Strain Characteristics of X80 Steels Under Hydrogen Environment

**Figure 7:**
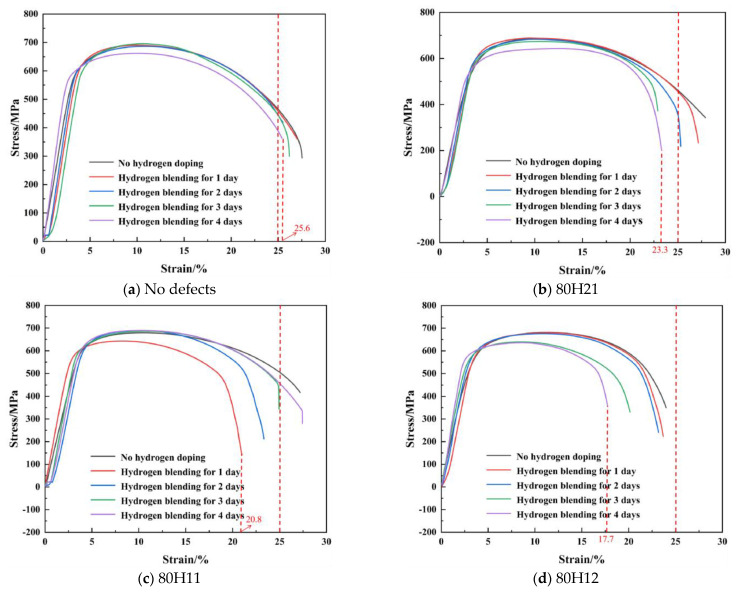
Stress–strain characteristics of X80 steel specimens with different defects and hydrogen concentrations.

We monitored the stress–strain characteristics of X80 steel specimens with varying defects and hydrogen concentrations during the tensile fracture process, as illustrated in Figure 7. Through comparison, it is evident that the strain of the fracture points for the specimens without defects can exceed 25%, regardless of hydrogen presence. The minimum strain observed at fracture point for a hydrogen charging time of 4 days is recorded at 25.6%. In contrast, the strains at fracture points for defective specimens (80H21, 80H11, and 80H12) are notably less than 25%, with a maximum strain of merely 23.8%. As depicted in Figure 7b–d, with an increasing hydrogen concentration, the fracture strain gradually decreases. A comparative analysis between Figure 7b,c reveals that higher hydrogen concentrations result in lower strains at fracture, suggesting that smaller defect diameters lead to more severe deterioration of the mechanical properties. Furthermore, through the comparison of Figure 7c,d, it is apparent that increased defect depths can also reduce the fracture strain.

### 3.3. Mechanical Properties of X80 Pipeline Steels

**Figure 8:**
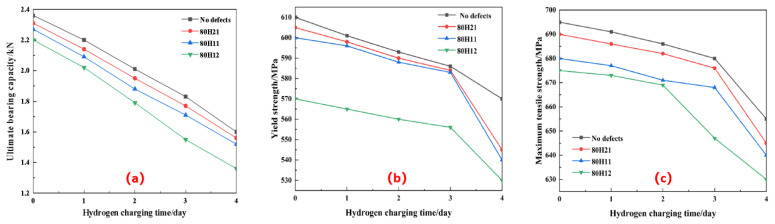
The mechanical properties of X80 pipeline steel under a hydrogen environment. (**a**) Ultimate bearing capacity, (**b**) Yield strength, (**c**) Ultimate tensile strength.

All specimens are subjected to tensile fracture tests on a WAW-1000 tensile testing machine to measure the ultimate bearing capacity (F), yield strength (σ_y_), and ultimate tensile strength (σ_u_), as illustrated in Figure 8a, 8b, and 8c, respectively. For specimens with the same defect, the ultimate bearing capacity, yield strength, and ultimate tensile strength decrease with the increase in the number of hydrogen charging cycles and hydrogen concentration. The greater the defect depth, the smaller the ultimate bearing capacity, yield strength, and ultimate tensile strength; the smaller the defect diameter, the smaller the three parameters as well. These findings indicate that for a hydrogen environment, the ultimate bearing capacity, yield strength, and ultimate tensile strength of X80 pipeline steel are closely related to corrosion defects and the hydrogen concentration.

### 3.4. Measurement Results for Weak Magnetic Signals

**Figure 9:**
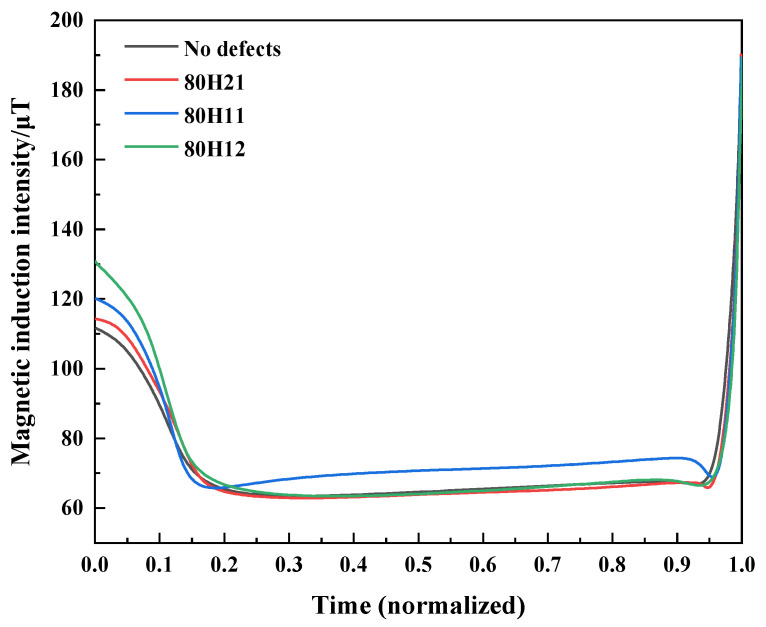
The curves of weak magnetic signals for original specimens with different defects.

The curves of weak magnetic signals for original specimens with different defects are illustrated in Figure 9. With the increase in defect depth, the initial values of axial magnetic signals become larger, where the values are 120.2 μT and 130.9 μT for 80H11 and 80H12, respectively. Conversely, with the increase in defect diameter, the initial values of axial magnetic signals decrease, where the values are 120.2 μT and 114.4 μT for 80H11 and 80H21. During the process of tensile fracture, weak magnetic signals initially decrease and then increase overall. The magnetic signals change more significantly in the initial stage (elastic stage), while minor fluctuations are observed in the later stages.

## 4. Discussion

### 4.1. Microscopic Morphology Analysis of Fracture Surface of X80 Pipeline Steels

**Figure 10:**
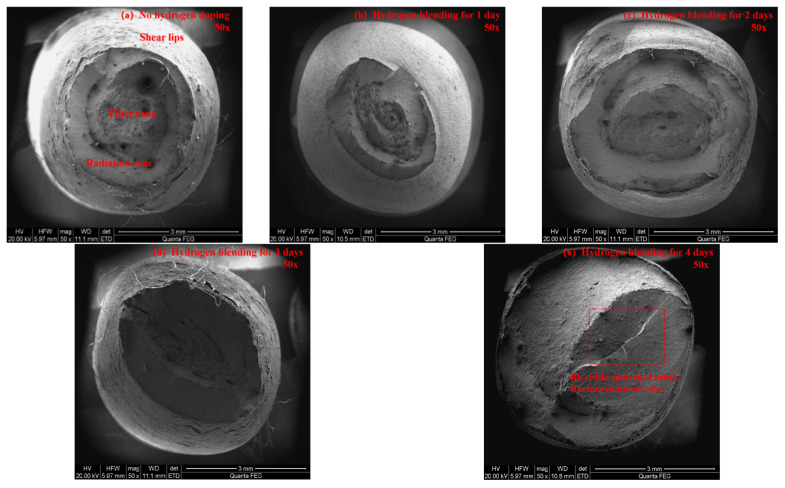
Microscopic morphology of the fracture surface of specimens at 50 times magnification.

**Figure 11:**
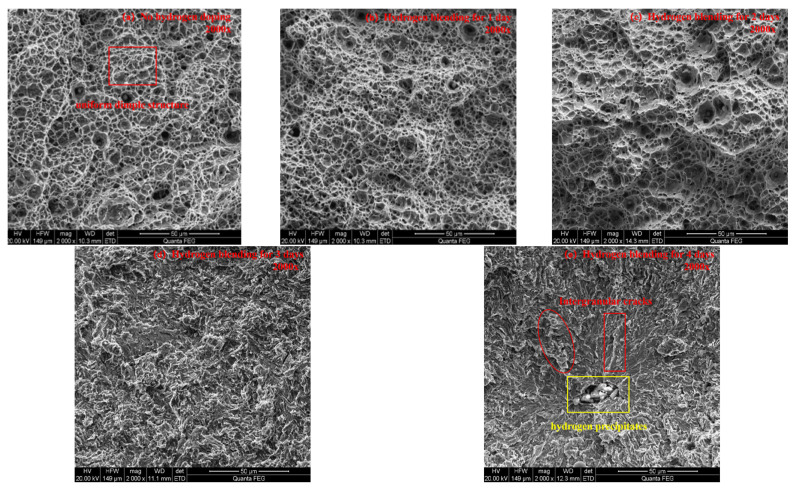
Microscopic morphology of the fracture surface of specimens at 2000 times magnification.

The microstructure of the fracture surface of X80 pipeline steel specimens is observed using a 200F field-emission environmental scanning electron microscope (200F FE-SEM). At magnifications of 50× and 2000×, the microstructure of the fracture surface is obtained for specimens without hydrogen charging and those charged with hydrogen for 1, 2, 3, and 4 days. Ultimately, the influence of the hydrogen charging time on the microstructure of the fracture surface is analyzed. Figure 10a shows the fracture surface morphology without hydrogen charging at a magnification of 50×. It is indicated that the fracture surface has obvious shear lips, fiber zones, and radiation zones. As the hydrogen charging time increases, the characteristics of the shear lips, fiber zones, and radiation zones gradually disappear. When the hydrogen charging time reaches 4 days (Figure 10e), a brittle fracture feature with a river-like pattern appears. Figure 11 shows the fracture surface morphology without hydrogen charging at a magnification of 2000×. It can be seen that the fracture surface has a uniform dimple structure. As the hydrogen charging time increases, the dimple features gradually become sparse and disappear. When the hydrogen charging time reaches 4 days (Figure 11e), brittle fracture features appear, such as intergranular cracks and hydrogen precipitates.

### 4.2. Weak Magnetic Characteristics of X80 Pipeline Steels Under Hydrogen Environment

**Figure 12:**
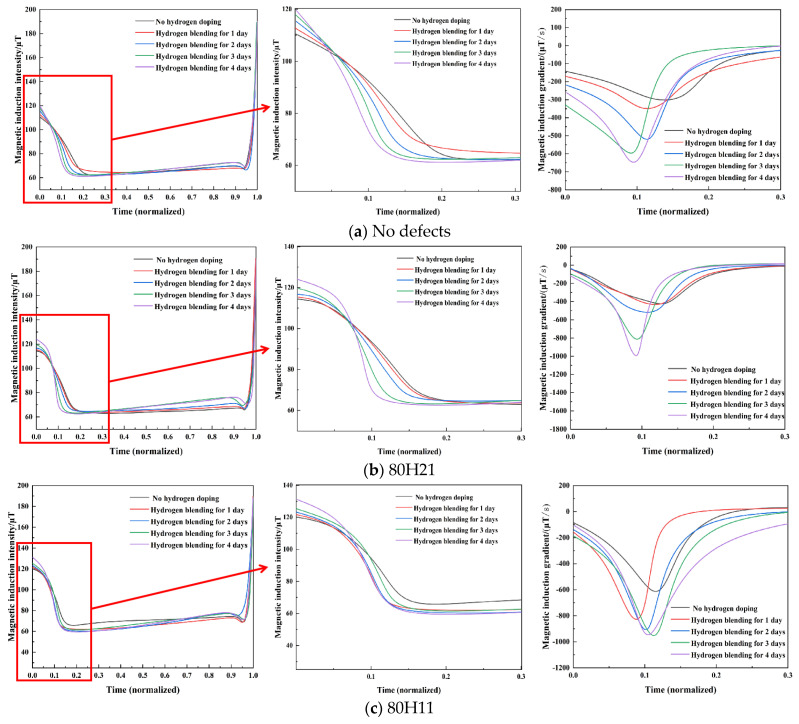

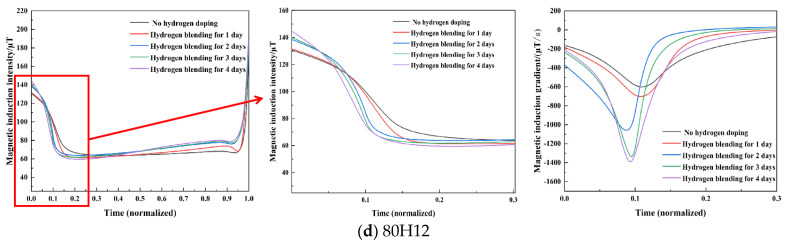
Weak magnetic signals of all the X80 pipeline steel specimens.

**Figure 13:**
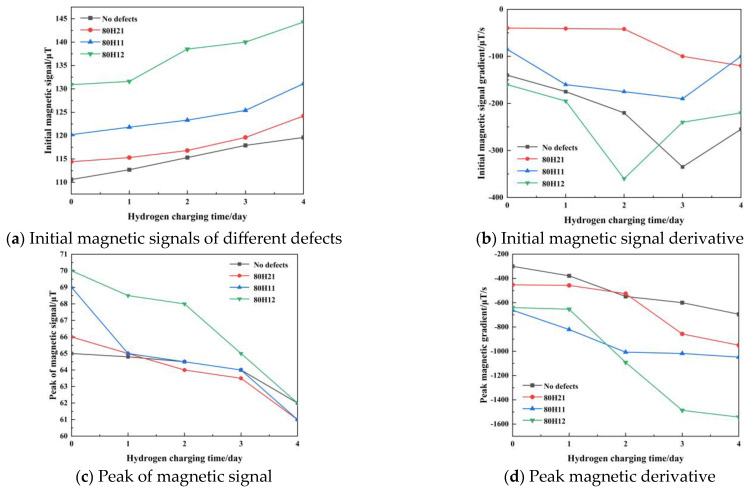
Four magnetic signal characteristics of all the X80 pipeline steel specimens.

To facilitate the comparison, the tensile time of the specimens is normalized. Weak magnetic signals of defect-free and defective specimens (80H21, 80H11, and 80H12) are presented in Figure 12. The variation in magnetic signals is the most obvious during the elastic phase of the tensile process. Therefore, magnetic signals are collected to observe the weak magnetic characteristics. Figure 13 presents the initial magnetic signal, the initial magnetic signal derivative, the peak of magnetic signal, and the peak magnetic signal derivative for all specimens. In Figure 13a, high hydrogen concentrations can cause larger initial magnetic signals, with values increasing as the defect depth increases, reaching a maximum of 144.3 μT. With the increase in defect diameter, the values of initial magnetic signals decrease, with a maximum of 131.1 μT. As shown in Figure 13c, the peak of magnetic signal gradually decreases with the increase in the hydrogen concentration. Additionally, it can be indicated from Figure 13d that increased hydrogen concentrations correlate with higher absolute values of peak magnetic signal derivatives. With an increasing defect depth and decreasing defect diameter, the absolute value of peak magnetic signal derivative gradually increases, achieving a maximum peak magnetic signal derivative of −1542.0 μT/s. Weak magnetic signals are related to the hydrogen concentration and corrosion defects, which can reflect the mechanical properties.

### 4.3. Critical Factor Analysis of Mechanical Properties

**Figure 14:**
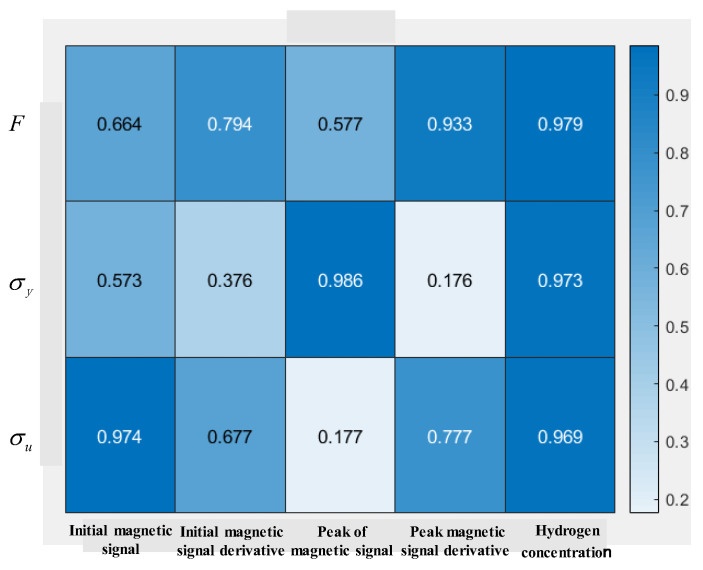
Correlation levels of mechanical properties and influencing factors.

To deeply explore the key factors influencing the ultimate bearing capacity, yield strength, and ultimate tensile strength of X80 pipeline steel, five influencing factors are selected, namely the initial magnetic signal, the initial magnetic signal derivative, the peak of magnetic signal, the peak magnetic signal derivative, and the hydrogen concentration. The original data of these parameters are shown in Table 3. The correlation levels calculated by gray correlation analysis are shown in Figure 14. The magnetic signal characteristics with the correlation level greater than 0.9 are selected as the critical influencing factors of the ultimate bearing capacity, yield strength, and ultimate tensile strength. As can be seen from Figure 14, the critical factors of ultimate bearing capacity are the peak magnetic signal derivative and hydrogen concentration; the critical factors of the ultimate tensile strength are the peak magnetic signal and hydrogen concentration; and the critical factors of the yield strength are the initial magnetic signal and hydrogen concentration.

### 4.4. Quantitative Assessment of F, σ_y_, and σ_u_

**Figure 15:**
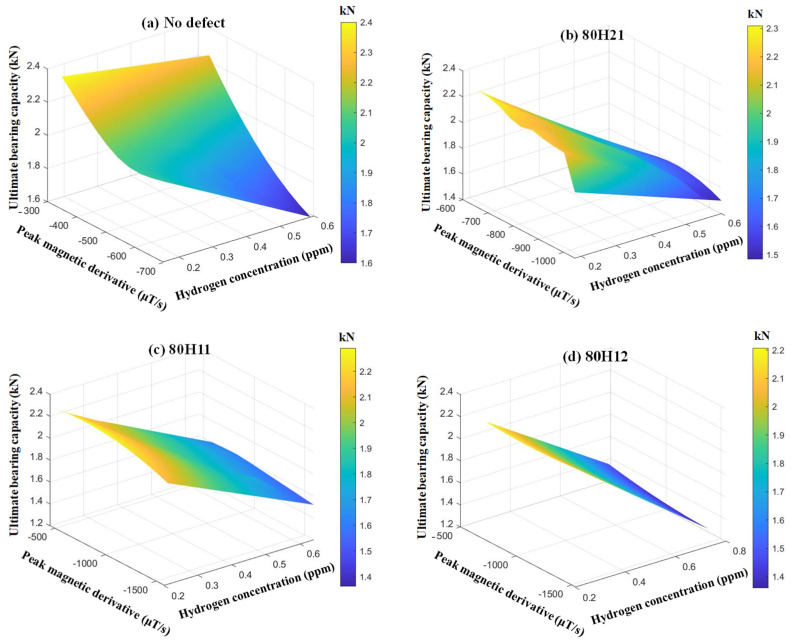
The relationship between F, C, and P of X80 steels with different defects.

Hydrogen corrosion of X80 steel can lead to a reduction in its mechanical properties. A key indicator called the ultimate bearing capacity F is selected to represent this effect. According to the correlation analysis in the previous text, weak magnetic signals can monitor the stress–strain characteristics during tensile fracture, thereby reflecting the ultimate bearing capacity F, and the feature of peak magnetic signal derivative is more obvious. For hydrogen environments, the hydrogen concentration C can affect the ultimate bearing capacity F and weak magnetic characteristics. Therefore, the ultimate bearing capacity F of X80 steel can be evaluated based on the hydrogen concentration C and peak magnetic signal derivative P. The relationship among the three for X80 steel with different defects is shown in Figure 15. For defect-free steel and defective steel (X80H21, X80H11, and X80H12), as the hydrogen concentration increases, the ultimate bearing capacity gradually decreases, and the absolute value of the peak magnetic signal derivative gradually increases. In addition, defect-free steel has the highest ultimate bearing capacity, which is 2.4 kN, followed by X80H21 at 2.3 kN and X80H11 at 2.25 kN, and X80H12 has the lowest at 2.2 kN. The quantitative relationship among F, C, and P for defect-free X80 steel is shown in Equation (1), with a goodness of fit R^2^ of 0.99.

**Figure 16:**
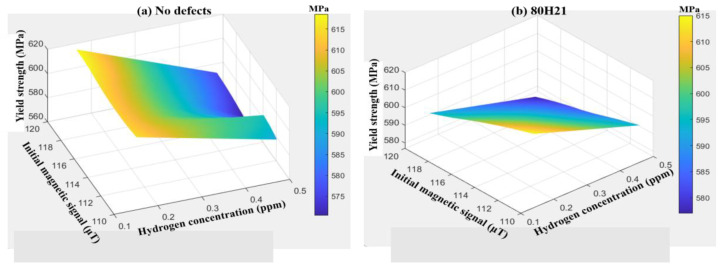

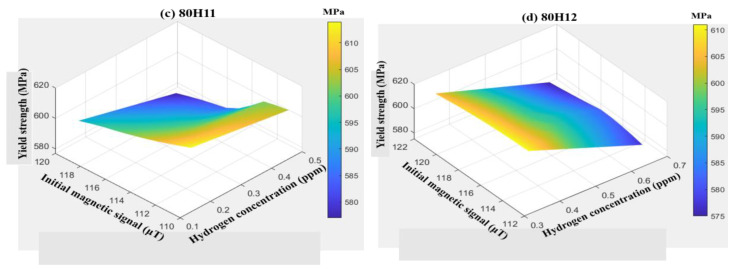
The relationship between σ_y_, C, and M_0_ of X80 steels with different defects.

**Figure 17:**
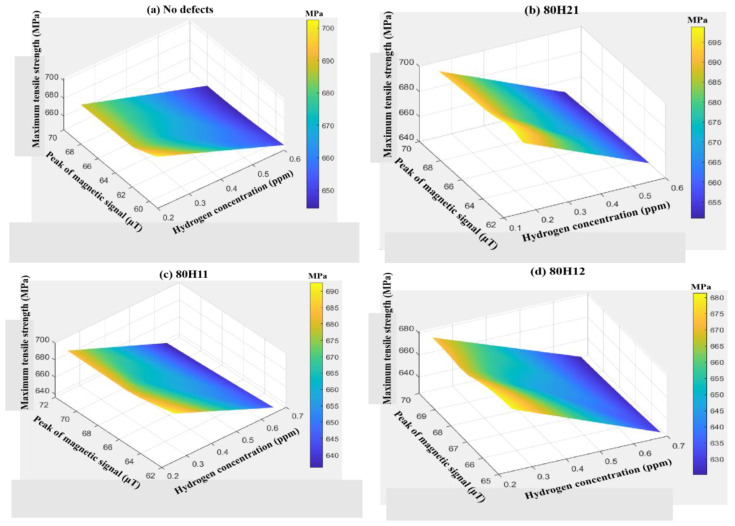
The relationship between σ_u_, C, and M_p_ of X80 steels with different defects.

Similarly, key indicators such as the yield strength σ_y_ and ultimate tensile strength σ_u_ can also be selected to demonstrate this influence. In Figure 16 and Figure 17, with the increase in hydrogen concentration, the yield strength and ultimate tensile strength gradually decrease, while the absolute values of the initial magnetic signal M_0_ and the peak magnetic signal M_p_ gradually increase. Similarly, the defect-free group has the highest yield strength and ultimate tensile strength. The quantitative relationship between σ_y_, C, and M_0_ of defect-free X80 steel is shown in Equation (2), with a goodness of fit R^2^ of 0.98. The quantitative relationship between σ_u_, C, and M_p_ is shown in Equation (3), with a goodness of fit R^2^ of 0.97.

F = 2.98 × 10^−7^ × C^2^ – 52 × P^2^ − 0.07 × C × P + 12.65 × C − 0.09 × P − 1.1 × 10^−4^ (1)

σ_y_ = −3.74 × C^2^ − 0.06 × M_0_^2^ − 0.57 × C × M_0_ + 0.9 × C + 13.57 × M_0_ − 90.75 (2)

σ_u_ = −60.25 × C^2^ + 0.24 × M_p_^2^ + 0.25 × C× M_p_ − 87.54 × C − 33.51 × M_p_ + 1865.46 (3)

## 5. Model Modification for Mechanical Properties Based on SSA

The quantitative relationship presented in Equation (1) can achieve the accurate evaluation of F for X80 steel without defects. However, the presence of defects can influence the quantitative relationship between F, C, and P under a hydrogen environment. Therefore, a model modification is carried out to dynamically update the quantitative relationship for X80 steels with different defects. Six coefficients (λ_1_–λ_6_) are introduced into Equation (1) for model modification, as shown in Equation (4).

F = λ_1_ × 2.98 × 10^−7^ × C^2^ − λ_2_ × 52 × P^2^ − λ_3_ × 0.07 × C × P + λ_4_ × 12.65 × C − λ_5_ × 0.09 × P − λ_6_ × 1.1 × 10^−4^ (4)

The values of six coefficients greatly influence the accuracy of quantitative relationship, but it is difficult to determine the coefficients directly. Therefore, it is necessary to optimize the six coefficients to improve the accuracy of assessment for ultimate bearing capacity. The SSA is mainly inspired by the foraging behavior and antipredator behavior of sparrows [32]. This method has the advantages of strong optimization ability and rapid convergence speed.

In the SSA, discoverers with favorable fitness values will prioritize obtaining food during the search process. In addition, the discoverer is responsible for finding food for the entire sparrow population and providing direction for all joiners to forage. Therefore, the discoverer can obtain a larger foraging search range than the joiner. The spotters’ location update formula is as follows [33]:

(5) $$X_{i,j}^{t+1}=\left\{\begin{array}{l} X_{i,j}^{t}\cdot \exp\left(\frac{-i}{\alpha \cdot Iter_{\max}}\right),\;R_{2}<ST\\ X_{i,j}^{t}+Q\cdot L,\;R_{2}\geq ST \end{array}\right.$$

where $X_{i,j}^{t}$ is the *i*-th sparrow in the *j* dimension under the current iteration *t*; *α* is a random number, *α*∈(0, 1]; *Iter_max_* is the maximum number of iterations; *R*_2_ and *ST* are the alert and safe values, respectively; *Q* is a random number, subject to standard normal distribution; and *L* is a d-dimensional matrix with one row, and all elements are 1.

The followers’ position is updated as follows [34]:

(6) $$X_{i,j}^{t+1}=\left\{\begin{array}{l} Q\cdot \exp\left(\frac{X_{worst}^{t}-X_{i,j}^{t}}{i^{2}}\right),\;i>\frac{n}{2}\\ X_{p}^{t+1}+\left|X_{i,j}^{t}-X_{p}^{t+1}\right|\cdot A^{+}\cdot L,\;i\leq \frac{n}{2} \end{array}\right.$$

where *X_worst_* is currently the worst position overall; n is the total number of sparrows, and when *i* > *n*/2, the *i*-th sparrow is very hungry; *X_p_* is the best place for spotters; and *A* is a one-row d-dimensional matrix with random elements of 1 or −1, *A^+^* = A^T^(AA^T^)^−1^.

Considering its own safety and ability to obtain food successfully, sparrows will select 10–20% of the individuals from the population for reconnaissance and alert, and the location update is as follows [35]:

(7) $$X_{i,j}^{t+1}=\left\{\begin{array}{l} X_{best}^{t}+\beta \left|X_{i,j}^{t}-X_{best}^{t}\right|,\;f_{i}>f_{g}\\ X_{i,j}^{t}+k\left(\frac{\left|X_{i,j}^{t}-X_{worst}^{t}\right|}{\left(f_{i}-f_{w}\right)+\varepsilon }\right),\;f_{i}=f_{g} \end{array}\right.$$

where *X_best_* is currently the best position overall; *β* is the step correction factor, subject to standard normal distribution; *f_i_* is the fitness of the sparrow at this time; *f_w_* and *f_g_*, respectively, represent the overall worst fitness and optimal fitness at this time; *k* is a random number, *k*∈(0, 1); and *ε* is an extremely small constant, *ε* = 10^−50^.

**Figure 18:**
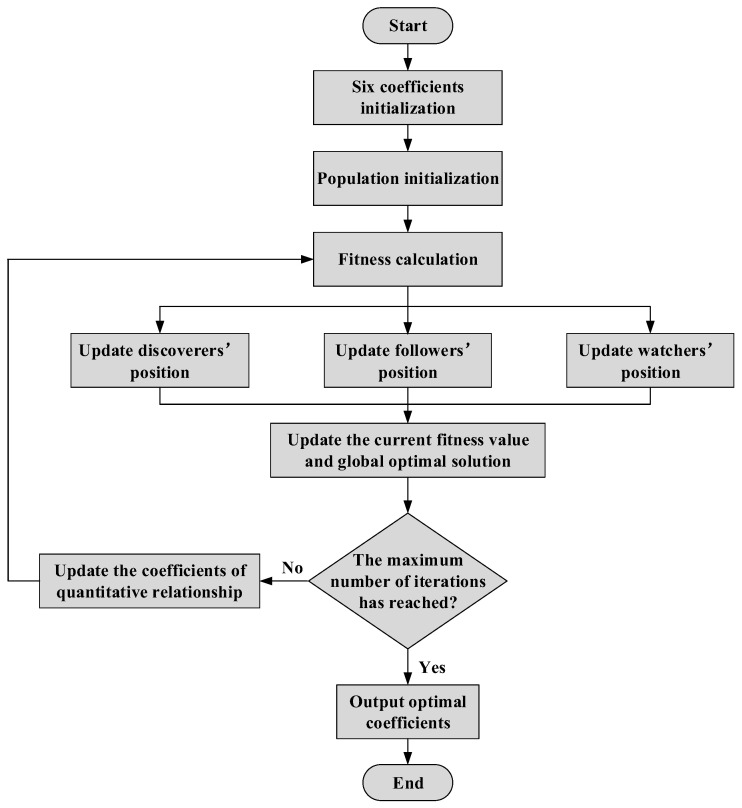
The flowchart of model modification based on the SSA.

The flowchart of model modification based on the SSA is shown in Figure 18. The population size is set to 30, the maximum number of iterations is 2000, and the warning value is 0.6. The ratios of spotters and followers are 0.7 and 0.3, respectively. The proportion of identified dangerous sparrows is 0.2. Six coefficients (λ_1_–λ_6_) are selected as the optimization variables, and the values of F, C, and P for X80 steels with different defects are substituted into Equation (2). And the mean relative error (MRE) of four groups of data is the fitness function, as shown in Equation (6). All coefficient values are constrained within the range of −1 to +1.

(8) $$G\left(X\right)=\min\sum_{i=1}^{n}\left(\left|\frac{F_{actual}-F_{calculate}}{F_{actual}}\right|\right)$$

Here, *F_actual_* is the actual value of the ultimate bearing capacity, and *F_calculate_* is the calculated value of the ultimate bearing capacity.

**Figure 19:**
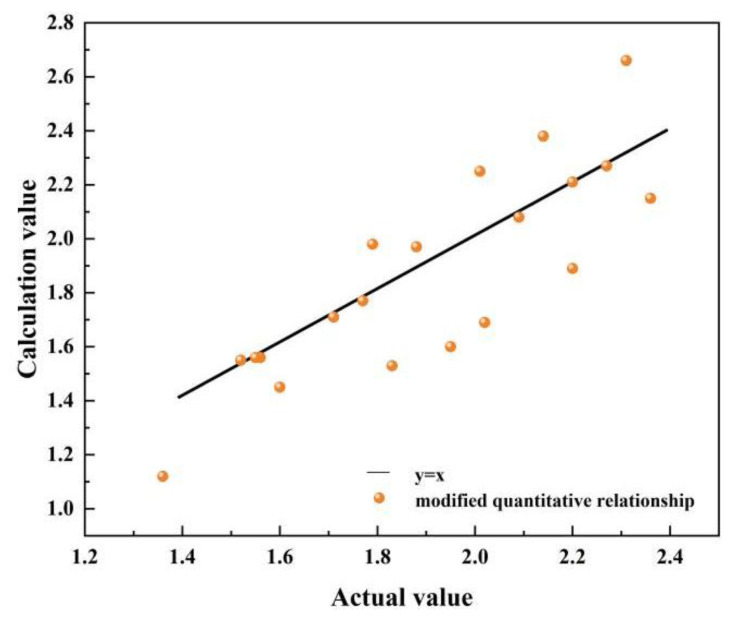
The scatter plot of F, calculated using modified quantitative relationship.

After model modification based on the SSA, the optimal values of the six coefficients are −0.013, 2.129 × 10^−8^, −0.034, −0.002, 0.046, and −0.006, respectively. The scatter plots of F calculated using the modified quantitative relationship are presented in Figure 19. It can be seen that the overall evaluation accuracy of the ultimate bearing capacity is high. Through calculation, the relative error between the ultimate bearing capacity of the experimental results and the modified quantitative relationship is 7.91% on average, which is within 10%, so it can meet the requirements of industrial applications. It demonstrates the feasibility of using weak magnetic technology to evaluate the ultimate bearing capacity of X80 steels under a hydrogen environment and different defects.

**Figure 20:**
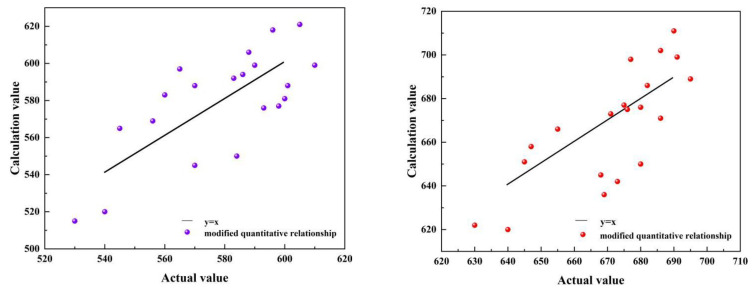
The scatter plots of σ_y_ and σ_u_, calculated using modified quantitative relationship.

Similarly, for Equations (2) and (3), after model modification using the SSA method, the scatter plots of σ_y_ and σ_u_ based on these modified quantitative relationships are presented in Figure 20. The relative errors of the yield strength and ultimate tensile strength remain within a margin of 16%, with MREs of 6.15% and 7.04%, respectively. The results demonstrate that the overall evaluation accuracy for both the yield strength and ultimate tensile strength is relatively high. These findings indicate that it is feasible to assess the yield strength and ultimate tensile strength of X80 steel under a hydrogen environment with various defects utilizing weak magnetic technology. For the established quantitative relationship, different working conditions of pipeline defects will correspond to specific coefficients, which can be applied for a high variety of defects in real pipelines.

## 6. Conclusions

In this study, a hydrogen corrosion experiment using H_2_S corrosion method is performed for X80 steels with different hydrogen charging times. Tensile fracture experiments of X80 steels with different defects and different hydrogen corrosion conditions are carried out, and weak magnetic technology is used to measure the magnetic signals during the tensile fracture process. Based on the correlation level, the quantitative relationships between the hydrogen concentration, magnetic signal characteristics, and mechanical properties are established, and the SSA is utilized to modify the quantitative relationship for X80 steels with various defects. The conclusions can be summarized as follows:

(1) The mechanical properties of X80 steel decrease with the increase in hydrogen concentration. The greater the defect depth, the smaller mechanical properties, while a reduction in the defect diameter will lead to a gradual decrease in mechanical properties.
(2) Microscopic analysis is performed on the tensile fracture specimens that are free of defects. The examination reveals that the fracture surface of the hydrogen-free samples exhibits characteristics such as shear lips, radiated zones, and fibrous regions, accompanied by a uniform dimple structure. As the duration of hydrogen charging is extended, the fracture surface of the samples displays brittle fracture characteristics. This observation indicates that the duration of hydrogen charging has a significant impact on the fracture properties of pipeline steel, leading to a transition from ductile to brittle fracture.
(3) An increased hydrogen concentration results in higher initial magnetic signals. The initial magnetic signal increases with the increase in defect depth and decreases with the increase in defect diameter; the peak value of the magnetic signal gradually decreases with the increase in hydrogen concentration; and an increased hydrogen concentration is related to the increase in the absolute value of the peak magnetic signal derivative. By calculating the degree of correlation, the critical factors of the ultimate bearing capacity are the peak magnetic signal derivative and hydrogen concentration; the critical factors of the ultimate tensile strength are the peak magnetic signal and hydrogen concentration; and the critical factors of the yield strength are the initial magnetic signal and hydrogen concentration.
(4) To ensure that these quantitative relationships can be extended to X80 pipeline steels with various defects, the model modification based on the SSA is performed. Through model modification, the average relative errors for the ultimate bearing capacity F, yield strength σ_y_, and ultimate tensile strength σ_u_ are found to be 7.91%, 3.15%, and 2.04%, respectively.

This paper proposes a quantitative method for evaluating the ultimate bearing capacity, yield strength, and ultimate tensile strength of X80 steels under a hydrogen environment using weak magnetic technology. However, there are still some limitations to the present study. In practical engineering, the defect shapes are non-uniform, and multiple defects may occur. Although the conversion of defect shapes into equivalent semi-elliptical defects is a widely accepted practice in pipeline integrity management, the quantitative evaluation method for mechanical properties under complex defect conditions is an important research direction in future. In addition, the application of quantitative evaluation method on the different steel grades needs to be further validated, which will be investigated in our future research.

## Figures and Tables

**Table 1:** Chemical composition of X80 steel.

C	Mn	Ni	Si	P	S	Cr	Cu
0.04–0.08%	≤1.7%	≤0.3%	≤0.55%	≤0.015%	≤0.005%	≤0.2%	≤0.25%

**Table 2:** Parameters of X80 steel specimens with different defects.

Parameter	Hydrogen Charging Time/Day	Parameter	Hydrogen Charging Time/Day	Parameter	Hydrogen Charging Time/Day	Parameter	Hydrogen Charging Time/Day
No defects	0	Diameter 1 mm Depth 1 mm (80H11)	0	Diameter 2 mm Depth 1 mm (80H21)	0	Diameter 1 mm Depth 2 mm (80H12)	0
	1		1		1		1
	2		2		2		2
	3		3		3		3
	4		4		4		4

**Table 3:** Some original data.

Initial Magnetic Signal	Initial Magnetic Signal Derivative	Peak of Magnetic Signal	Peak Magnetic Signal Derivative	Hydrogen Concentration	F	σ_u_	σ_y_
110.6	−140	65	−300.6	0.16	2.36	695	610
112.7	−170	70	−378.6	0.25	2.2	691	601
115.3	−220	65	−548.8	0.4	2.01	686	593
117.9	−255	64	−600	0.45	1.83	680	586
119.6	−340	62	−695.1	0.55	1.6	655	570

## Data Availability

The data presented in this study are available on request from the corresponding author.
